# Methods: Aortic wall deformation assessment by ultrafast ultrasound imaging: Application to bicuspid aortic valve associated aortopathy

**DOI:** 10.3389/fphys.2023.1128663

**Published:** 2023-03-03

**Authors:** Guillaume Goudot, Charles Cheng, Alexis F. Guédon, Tristan Mirault, Olivier Pedreira, Alexandre Dahan, Louise Z. Wang, Mathieu Pernot, Emmanuel Messas

**Affiliations:** ^1^ Physics for Medicine Paris, INSERM U1273, ESPCI Paris, CNRS FRE, PSL Research University, Paris, France; ^2^ Vascular Medicine Department, Georges-Pompidou European Hospital, AP-HP, Université Paris Cité, Paris, France; ^3^ Université Paris Cité, INSERM U970, PARCC, Paris, France

**Keywords:** ultrasound, stiffness, bicuspid aortic valve, aorta, echocardiogaphy

## Abstract

**Purpose:** Aortic maximal rate of systolic distention (MRSD) is a prognosis factor of ascending aorta dilatation with magnetic resonance imaging. Its calculation requires precise continuous tracking of the aortic diameter over the cardiac cycle, which is not feasible by focused ultrasound. We aimed to develop an automatic aortic acquisition using ultrafast ultrasound imaging (UUI) to provide access to the aortic MRSD.

**Methods:** A phased array probe and developed sequences at 2000 frames/s were used. A created interface automatically tracked the anterior and posterior aortic walls over the cardiac cycle. Tissue Doppler allowed a precise estimation of the walls’ movements. MRSD was the maximum derivative of the aortic diameter curve over time. To assess its feasibility, 34 patients with bicuspid aortic valve (BAV) and 31 controls were consecutively included to evaluate the BAV-associated aortopathy at the sinus of Valsalva, the tubular ascending aorta, and the aortic arch.

**Results:** UUI acquisitions and the dedicated interface allow tracking of the aortic diameter and calculating the MRSD for the BAV patients and controls (mean age of 34 vs. 43 years, *p* = 0.120). A trend toward lower deformation in the different aortic segments was observed, as expected. Still, only the MRSD with UUI was significantly different at the sinus of Valsalva in this small series: (0.61 .10^3^.s^–1^ [0.37–0.72] for BAV patients vs. 0.92 .10^3^.s^–1^ [0.72–1.02] for controls, *p* = 0.025).

**Conclusion:** Aortic deformation evaluated with UUI deserves attention with a simple and automated measurement technique that could assess the segmental aortic injury associated with BAV.

## 1 Introduction

Bicuspid aortic valve (BAV) associated aortopathy refers to all structural changes in the ascending thoracic aorta, resulting in a higher risk of aortic aneurysm and acute complications, such as aortic dissection or aneurysm rupture. BAV is the most common cardiac malformation, estimated at 1% of the general population, and the risk of developing an aneurysm of the ascending aorta is high in this specific population (Braverman, 2011; Michelena et al., 2021). Clinical management of the BAV-associated aortopathy is still difficult, with an uncertain risk of aortic dilatation progression, although new imaging markers of progression are emerging. Using new biomechanical markers, such as aortic stiffness and hemodynamic variations of the aortic flow, may be useful as prognostic markers (Nistri et al., 2008; Tierney et al., 2018; Goudot, et al., 2019a; Longobardo et al., 2021; Michelena, 2022). In the case of BAV, there is indeed an early and segmental aortic remodeling, resulting in a progressive disappearance of the elastic plates and an increase in the collagen content of the extracellular matrix (Goudot, et al., 2019b). The evaluation of aortic deformation is complex with conventional ultrasound because of the difficulty of accurately following the aortic walls and visualizing an extended aortic portion within the same picture. Magnetic resonance imaging (MRI) can improve aortic wall tracking, even though with a low frame rate, and Aquaro et al. proposed new prognostic markers for aortic dilation, such as the Maximal Rate of Systolic Distention (MRSD) of the ascending aorta, derived from the aortic diameter variation curve over the cardiac cycle (Aquaro et al., 2013). This marker appears to be better able to identify early stiffness abnormalities and to predict subsequent aortic dilatation (Aquaro et al., 2017). Ultrasound imaging also can finely track the aortic wall, with a much higher frame rate, and thus lead to the development of more sensitive markers than B-mode diameter variation. Through the development of Ultrafast Ultrasound Imaging (UUI), a high image rate makes it possible to track the ultrasound wall in ultrafast tissue Doppler (Tanter et al., 2002; Papadacci et al., 2019). Further use of a phased-array probe now allows access to the wall of the ascending aorta. Translating such markers usable in echocardiography would allow easy access to these markers during follow-up echocardiography, regularly performed in the case of BAV, because it allows a quick, inexpensive, and non-irradiating follow-up of the functions of the left ventricle, the aortic valve, and the ascending aorta. In this work, our objective was to develop an automated aortic deformation collection in UUI and apply it to a series of patients with BAV compared to normal first-degree relatives.

## 2 Methods

### 2.1 Population

This is a cross-sectional study of 65 consecutive patients undergoing dedicated consultation between January 2019 and July 2020 at the European hospital Georges-Pompidou, a reference center for BAV disease. First-degree healthy relatives, i.e., with a tricuspid aortic valve, also screened for BAV, were used as controls. The ethical committee approved this study, and patients signed a written informed consent form. Confirmation of BAV was retained in the case of the short-axis view of the aortic valve with the presence of only two functional cusps.

### 2.2 Transthoracic cardiac ultrasound

Transthoracic echocardiography was performed using commercially available equipment (IU22^®^; S5-1, 1–5 MHz, 80 elements probe; Philips Medical Systems^©^, Andover, MA). Analysis of the aortic valve and the ascending aorta was systematically performed following a dedicated protocol previously published (Goudot, et al., 2019a). The thoracic aorta was assessed at three segments of interest, each time in a longitudinal section with registration and alignment using B mode: The sinus of Valsalva, the initial portion of the ascending aorta, following the aortic valve, using parasternal long-axis view. The diameter measurement was performed from sinus base to sinus base (maximum diameter); then the ascending tubular aorta, more than 1 cm from the sinotubular junction, corresponding to a zone of parallel aortic walls; And the initial portion of the aortic arch, this time in supra-sternal view.

### 2.3 Aortic ultrafast ultrasound imaging

**FIGURE 1:**
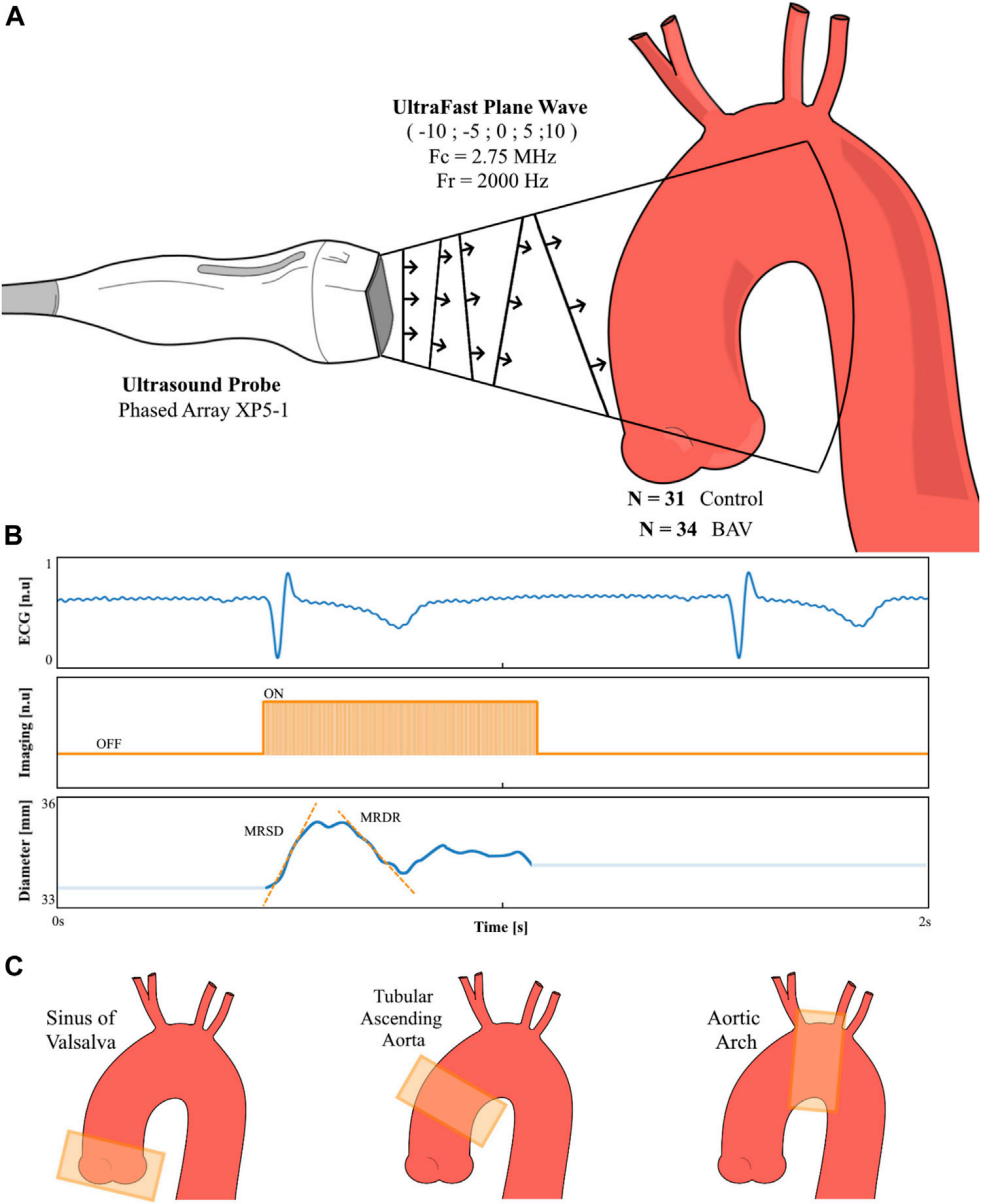
Technical methodology overview. A plane wave sequence with five different angles have been adapted for a phased array probe (1–5 MHz frequency). Starting from a conventional B-mode ultrasound allowing the placement of the probe in front of the aortic segment to be imaged **(A)**, the plane’s waves’ acquisition was launched at the foot of the QRS. The aortic Diameter is then recorded with the calculation of the Maximal Rate of Systolic Distension (MRSD) and Maximal Rate of Diastolic MRDR **(B)**. This procedure was repeated on three segments of the aorta **(C)**: sinus of Valsalva, tubular ascending aorta, and aortic arch; in 34 patients with bicuspid aortic valve (BAV) and 31 healthy relatives (Controls).

Evaluation of the ascending aorta was performed using an Aixplorer^©^ device (Supersonic Imagine^©^. Aix-en-Provence. France), with a phased-array probe (2.75 MHz canter frequency. 96 elements. SuperSonic Imagine^©^). After alignment of the ascending aorta according to the sections specified above, the acquisitions were performed using plane waves with five angles (−10; −5; 0; 5; 10), and a frame rate of 2.000 s^−1^. The acquisition’s duration was 507 ms and was triggered at the start of the QRS. The study protocol is synthesized in Figure 1.

**FIGURE 2:**
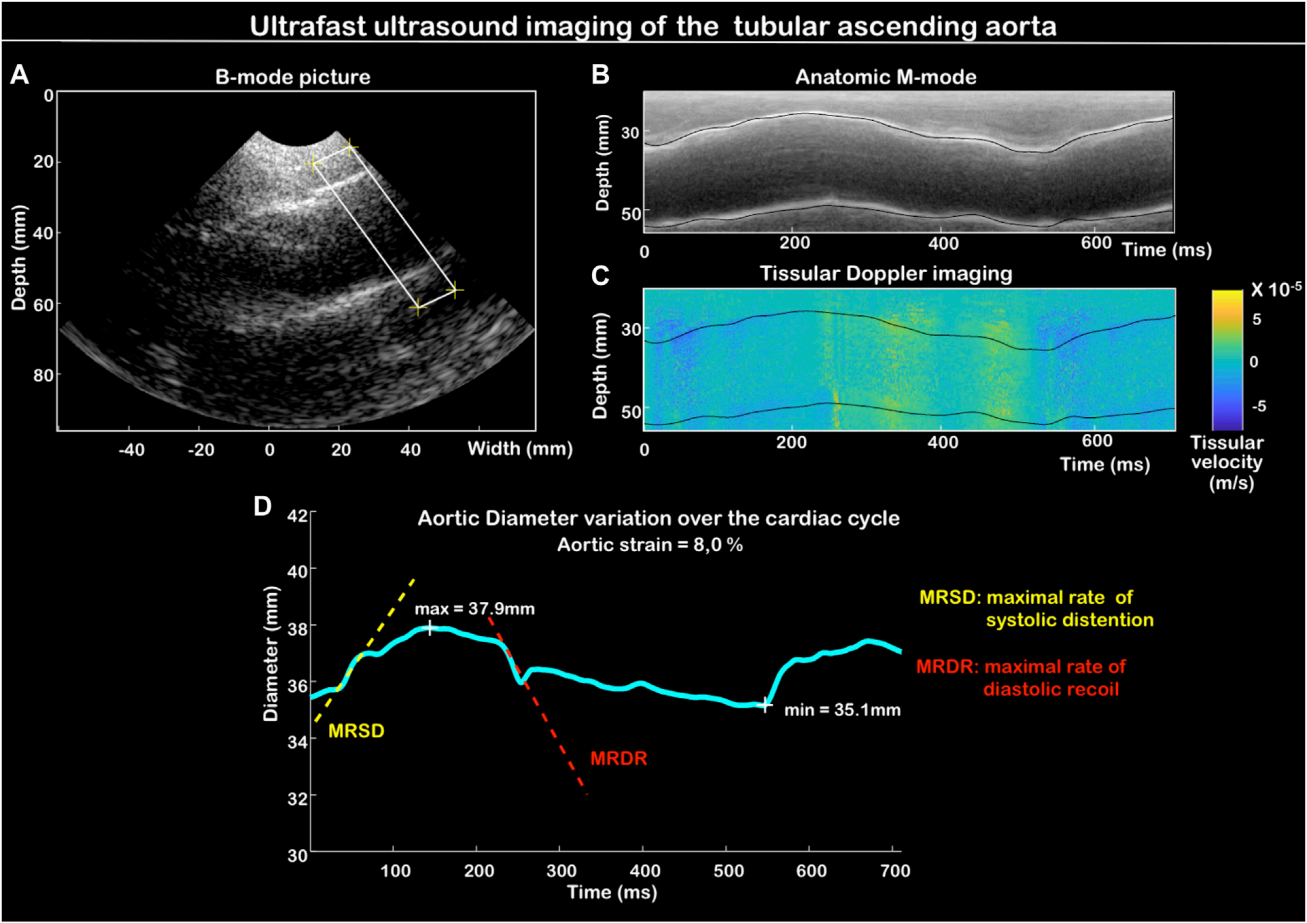
Aortic distensibility measurement method: delimitation of the area of interest of the aorta on the B-mode image. Here at the level of the sinus of Valsalva **(A)**. Automated wall delineation from the anatomic M-mode (TM image) of the delineated segment **(B)**. Collecting tissue velocity values (Tissue Doppler imaging) for each aortic wall over time (time) **(C)**. Determination of the aortic diameter variation curve over time **(D)**.

**FIGURE 3:**
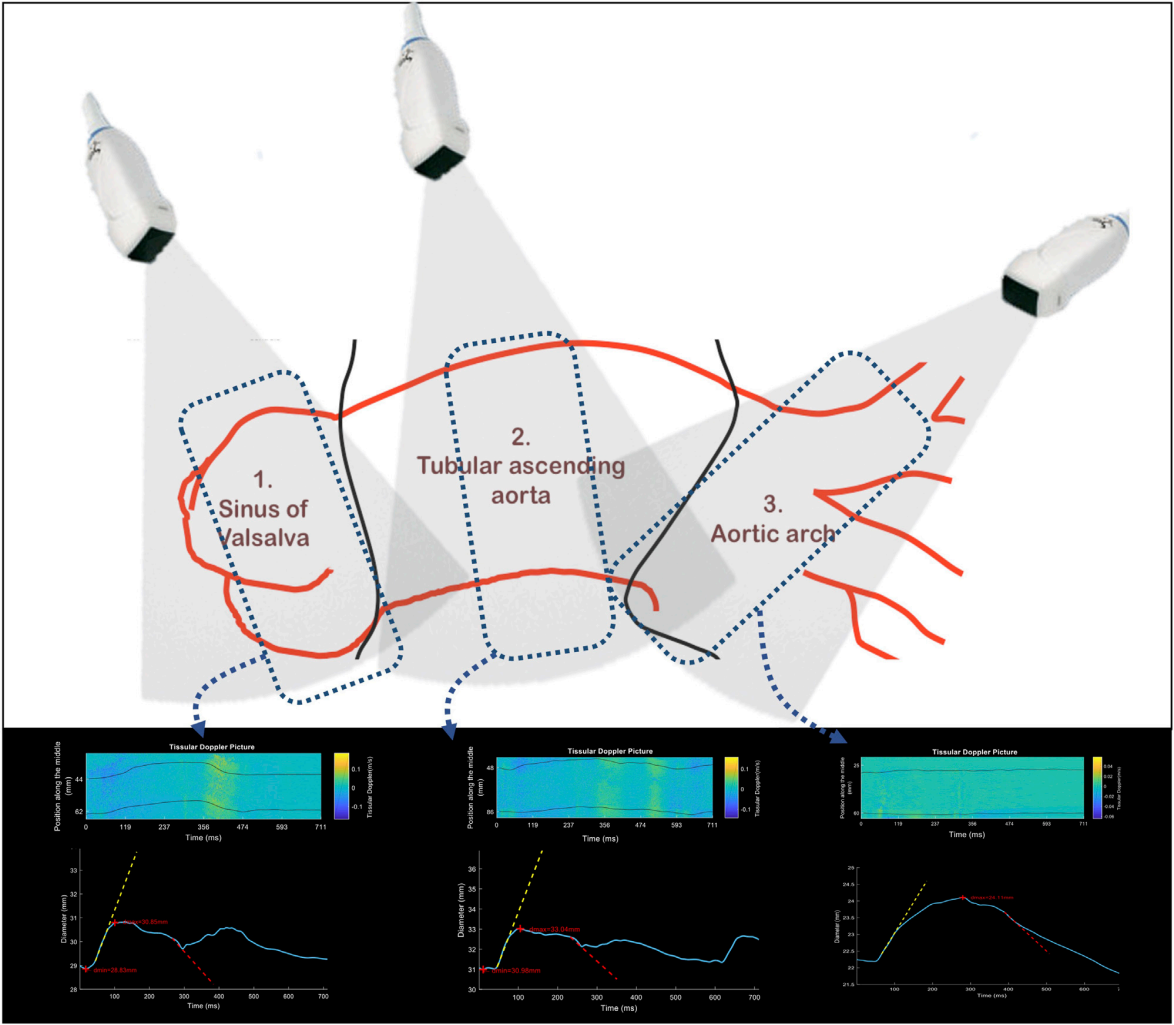
Schematic positioning of the echocardiography probe in front of each segment of the ascending thoracic aorta: sinus of Valsalva, ascending tubular aorta, and aortic arch. At the level of each segment is represented an example of tissue Doppler collection, with the representation of the diameter curve (blue curve) with the maximum positive (MRSD, yellow) and maximum negative (MRDR, red) slopes.

Compared to carotid arterial wall imaging, signal analysis of the aortic wall is much more complex due to the significant movement of the aorta during the cardiac cycle. For this reason, we have implemented automated monitoring of the aortic wall. From the reconstructed B-mode ultrasound image (Figure 2A), we delineate the axis of the aorta to obtain a TM (time-movement) representation of the aortic walls over time (Figure 2B), like the TM-anatomical mode proposed in conventional imaging, to overcome the orientation of the probe (Carerj et al., 2003). From the resulting image, the walls could then be automatically delineated with the possibility of manual correction. Due to the inaccuracy of the TM-anatomical mode for evaluating fine variations in aortic diameter, we used this sequence only for wall location. From the anterior and posterior wall position coordinates, the tissue Doppler data contained in the same UUI acquisition were extracted and allowed the calculation of the velocity of each aortic wall. The variation in aortic diameter was obtained by calculating the difference in tissue velocity between the two walls over time (Figure 3). The presented aortic diameters are measured in diastole (minimal diameter, mm). The aortic strain is the variation of the aortic diameter during the cardiac cycle (%) according to the formula (Dmax–Dmin)/Dmin with Dmax the maximum aortic systolic diameter (mm), Dmin, the minimal diastolic diameter (mm). Aortic wall distensibility (mmHg^-1^) was measured as 2(Dmax–Dmin)/[Dminx(SBP–DBP)] with SBP the systolic blood pressure (mmHg), and DBP the diastolic blood pressure (mmHg). MRSD and MRDR are, respectively, positive and negative peaks in the derivative of the aortic diameter curve over the cardiac cycle, normalized as a percentage of the diastolic aortic diameter, and are presented in s^-1^.

### 2.4 Statistical analysis

Continuous variables are presented by the median [25^th^–75^th^ percentiles]. Because of the small number of patients, the lack of compliance with the conditions for testing for comparability of means (normal distribution and homoscedasticity of variances), a Wilcoxon test was used for paired data comparisons. The correlation was performed using a Spearman rank test. The interclass correlation coefficient (ICC) assessed the reliability of VI. Statistical significance was considered at the 0.05 level. Analyses were performed using R^®^ software (R-Studio^®^, Boston, MA, United States).

## 3 Results

### 3.1 Population

Thirty-four patients with BAV and 31 controls were included prospectively. Patient characteristics are described in Table 1. As anticipated, greater aortic dilatation was observed, particularly at the level of the sinus of Valsalva, the only significant difference between the two groups (30.6 mm in case of BAV [26.4–33.5] vs. 25.5 mm [23.7–28.1], *p* = 0.048). The values of systolic and diastolic blood pressure essential for the interpretation of arterial stiffness indicators were not significantly different between the groups.

**TABLE 1:** Characteristics of BAV patients and controls. BAV: bicuspid aortic valve; DBP: diastolic blood pressure; SBP: systolic blood pressure; PP: pulse pressure (SBP–DBP). *p*-values are obtained from the Mann-Whitney test. Data in bold correspond to statistically significant comparisons (*P* < 0.05).

	BAV patients	Controls	*p*-value
	N = 34	N = 31	
Age (Year)	34 [25–55]	43 [26–55]	0.120
Men (%)	24 (70,6)	23 (74,2)	0.750
Sinus of Valsalva diameter (mm)	30.6 [26.4–33.5]	25.5 [23.7–28.1]	**0.048**
Tubular ascending diameter (mm)	31.6 [27.9–35.2]	27.5 [24.6–30.2]	0.060
Aortic arch diameter (mm)	25.9 [21.7–29.5]	27. 0 [23.9–29.8]	0.438
SBP (mmHg)	121 [110–130]	118 [111–120]	0.409
DBP (mmHg)	69.9 [63.5–76.2]	61.5 [59.0–69.0]	0.089
PP (mmHg)	49 [43–58]	51 [50–60]	0.126

### 3.2 Semi-automatic measurement of aortic deformation by UUI

Intraclass ICC was good at 0.76. Interclass was moderate at 0.73. In the case of repeated analysis over three acquisitions (3 cardiac cycles), the inter-observer reproducibility increased with an ICC of 0.86, thus allowing good reproducibility.

### 3.3 Aortic deformation between patients with BAV and controls

Diameter measurement results and aortic deformation parameters are presented in Table 2. Of the small number evaluated, there was no significant difference in diameter at each segment of the ascending aorta. i.e., at the sinus of Valsalva, the tubular ascending aorta, and the aortic arch. We found a smaller variation in aortic diameter (aortic strain) at each aortic segment, even though without significant difference between the two groups. MRSD and MRDR values followed the same trend, with consistently lower values in BAV patients. The difference in MRSD at the sinus of Valsalva was the largest, with a significant difference between the two groups (0.61 .10^3^ s^-1^ [0.37–0.72] for BAV patients vs. 0.92 .10^3^ s^-1^ [0.72–1.02] for controls. *p* = 0.025).

**TABLE 2:** Aortic parameters obtained by ultrafast ultrasound imaging for BAV patients and controls. Comparisons are made using a Mann-Whitney test. BAV: bicuspid aortic valve; MRSD: maximal rate of systolic distension. A Mann-Whitney test obtains the *p*-values. Data in bold correspond to statistically significant comparisons (*P* < 0.05).

Aortic level	Aortic deformation parameters	BAV patients	Controls	*p*-value
		N = 34	N = 31	
Sinus of Valsalva	Strain (%)	8.2 [7.01–10.12]	9.67 [6.9–11.5]	0.472
	Distensibility (10^−3^.mmHg^–1^)	3.49 [2.78–4.83]	4.84 [3.20–7.32]	0.275
	MRSD (10^3^.s^–1^)	0.61 [0.37–0.72]	0.92 [0.71–1.02]	**0.025**
	MRDR (10^3^.s^–1^)	-0.62 [-0.74 to -0.50]	-0.61 [-0.95 to -0.50]	0.493
Tubular ascending aorta	Strain (%)	7.9 [7.0–10.0]	9.0 [8.0–12.5]	0.366
	Distensibility (10^-3^.mmHg^–1^)	1.61 [1.32–2.64]	1.86 [1.22–2.00]	0.502
	MRSD (10^3^.s^–1^)	0.97 [0.75–1.07]	1.05 [0.87–1.18]	0.560
	MRDR (10^3^.s^–1^)	-0.70 [-0.96 to -0.55]	-0.72 [-0.90 to -0.58]	0.864
Aortic arch	Strain (%)	7.0 [4.5–10.0]	7.2 [4.9–8.9]	0.870
	Distensibility (10^-3^.mmHg^–1^)	1.23 [0.63–1.66]	1.36 [0.84–2.42]	0.483
	MRSD (10^3^.s^–1^)	0.99 [0.80–1.05]	0.91 [0.79–1.13]	0.734
	MRDR (10^3^.s^–1^)	-0.54 [-0.65–0.36]	-0.75 [-0.94 to -0.55]	**0.029**

## 4 Discussion

To our knowledge, this work is the first study to evaluate the ascending aorta by transthoracic ultrafast ultrasound imaging. In this work, we demonstrated the feasibility of UUI using a phased array probe for reliable measurements of aortic deformation. By developing an automated wall-tracking system, we have created a rapid measurement of circumferential stiffness parameters in a single cardiac cycle. From a pilot study using a small series of patients with BAV, we have found a segmental increase of stiffness preferentially at the level of the sinus of Valsalva by using the MRSD measurement, which is not available in conventional ultrasound. This technique represents a significant advance in aortic wall imaging as the extraction of tissue Doppler data allows much greater accuracy than simple B-mode signal tracking of the arterial wall. This technique has already demonstrated robustness in arterial wall imaging with a linear probe. The use of a phased array probe represented an additional difficulty. Still, it was mandatory, given the need for positioning the probe between two ribs and the depth of the aorta in transthoracic imaging. By obtaining the diameter variation curve over time, we were thus able to get MRSD values at each segment of the ascending aorta. This method could therefore be easily added to the follow-up of BAV patients. in whom an ultrasound evaluation of aortic diameters is already recommended (Erbel et al., 2014). While there are few studies on the prognostic role of these indicators of aortic stiffness, Aquaro et al. have demonstrated the value of measuring MRSD in the ascending aorta by MRI in assessing the risk of subsequent aortic dilatation (Aquaro et al., 2017). The evaluation of similar morphological markers by transthoracic ultrasound has the advantage of a simple and easily accessible measurement, which can be easily integrated into the follow-up of patients with BAV or with an ascending thoracic aortic aneurysm usually performed by echocardiography and until now limited to the measurement of aortic diameters (Erbel et al., 2014). If evaluating an aortic deformation marker such as the MRSD allows a rapid clinical translation, this methodology must be placed in perspective by the complexity of the hemodynamic variations in the ascending aorta and the blood flow-aortic wall interactions. Aortic wall alterations are indeed at least partly related to the flow constraints applied (Bollache et al., 2018). The use of wall shear stress measurement, as shown in 4D flow MRI (Guala et al., 2022; Kiema et al., 2022; Soulat et al., 2022; Qin et al., 2023), is thus particularly useful, although yet to be available with echocardiography. An appealing prospect is the use of ultrasound innovations in the field flow mapping to combine aortic wall markers, such as stiffness, and wall shear stress, to appreciate better the causative phenomena of aortic remodeling and aneurysmal progression (Cai et al., 2019; Kainuma et al., 2022).

### 4.1 Limitation

All the recovered data were processed remotely because the evaluation of distensibility parameters is unavailable on the commercial UUI ultrasound device. An automated method for real-time collecting stiffness parameters could nevertheless be quickly implemented. The objective of this work was to evaluate the feasibility of UUI aortic deformation measurements and not their prognostic role. Caution should be observed when interpreting statistical comparisons in our cohort. Because of the small number of patients in this initial exploratory work, only non-significant trends in distensibility differences were found for the different aortic segments. Data from the literature converge towards an increase in the global stiffness of the ascending aorta in the case of BAV, demonstrated in a larger cohort of patients (Nistri et al., 2008). We cannot appreciate all the heterogeneity of BAV cases; in particular, the influence of the anatomy of the aortic valve is not possible here because the great majority of cases with 1LR BAV, according to the Sievers classification (Sievers and Claudia, 2007). We did not perform longitudinal follow-ups of patients, preventing any conclusion on the potential prognostic role of aortic deformation or stiffness indicators. This evaluation is part of a subsequent study, currently being conducted by multimodal imaging, including two evaluations 2 years apart by MRI, conventional ultrasound, and UUI (NCT03474159).

## 5 Conclusion

Ultrafast ultrasound imaging allows simultaneous automated evaluation of aortic deformation parameters over the cardiac cycle. The application of this measurement technique on the ascending aorta appears to be a simple, easily accessible, and reliable technique for assessing deformation as a potential prognostic tool for subsequent dilatation.

## Data Availability

The original contributions presented in the study are included in the article/supplementary materials, further inquiries can be directed to the corresponding author.
